# Biogeographic parallels in thermal tolerance and gene expression variation under temperature stress in a widespread bumble bee

**DOI:** 10.1038/s41598-020-73391-8

**Published:** 2020-10-13

**Authors:** Meaghan L. Pimsler, Kennan J. Oyen, James D. Herndon, Jason M. Jackson, James P. Strange, Michael E. Dillon, Jeffrey D. Lozier

**Affiliations:** 1 Department of Biological Sciences, The University of Alabama, Tuscaloosa, AL 35487 USA; 2 Department of Zoology and Physiology and Program in Ecology, University of Wyoming, Laramie, WY 82071 USA; 3 Department of Biological Sciences, University of Cincinnati, Cincinnati, OH 45221 USA; 4 USDA-ARS Pollinating Insects Research Unit, Utah State University, Logan, UT 84322 USA; 5 Department of Entomology, The Ohio State University, Columbus, OH 44691 USA

**Keywords:** Genetic variation, Evolution, Physiology

## Abstract

Global temperature changes have emphasized the need to understand how species adapt to thermal stress across their ranges. Genetic mechanisms may contribute to variation in thermal tolerance, providing evidence for how organisms adapt to local environments. We determine physiological thermal limits and characterize genome-wide transcriptional changes at these limits in bumble bees using laboratory-reared *Bombus vosnesenskii* workers. We analyze bees reared from latitudinal (35.7–45.7°N) and altitudinal (7–2154 m) extremes of the species’ range to correlate thermal tolerance and gene expression among populations from different climates. We find that critical thermal minima (CT_MIN_) exhibit strong associations with local minimums at the location of queen origin, while critical thermal maximum (CT_MAX_) was invariant among populations. Concordant patterns are apparent in gene expression data, with regional differentiation following cold exposure, and expression shifts invariant among populations under high temperatures. Furthermore, we identify several modules of co-expressed genes that tightly correlate with critical thermal limits and temperature at the region of origin. Our results reveal that local adaptation in thermal limits and gene expression may facilitate cold tolerance across a species range, whereas high temperature responses are likely constrained, both of which may have implications for climate change responses of bumble bees.

## Introduction

Understanding organismal responses to abiotic extremes has become a pressing concern given ongoing and projected changes in environmental conditions^1,2^. Species with large geographic distributions can encounter substantial environmental heterogeneity^3,4^ and are excellent models for illuminating the evolution of physiological and genetic variation related to temperature. The degree to which individuals are adapted to regional conditions or tolerate a range of abiotic variation via flexibility or plasticity is of particular interest^5^, and how selection has shaped range-wide responses to abiotic stress through molecular mechanisms such as gene expression regulation has received increasing attention^6–10^. Linking physiology and transcriptomics can thus be a valuable approach for teasing apart how species with large ranges adapt to local variation in their environment^5^.

Gradients in ambient temperature drive numerous biogeographic phenomena^5,11^, including contemporary range dynamics under global climate change^11^. Thermal tolerance, or the critical thermal limits (CTLs) of organismal performance at high (CT_MAX_) and low (CT_MIN_) temperatures^12^, is one metric that may be shaped by selection to climate variation^5^. However, the strength of correlations between climate and CTLs differ at high and low temperatures^11,13^ due to differences in the relative selective importance of high or low temperature extremes^14^. For example, studies have revealed that many terrestrial ectotherms show little variation in CT_MAX_ but pronounced differences in CT_MIN_ with geographic temperature gradients^14–16^. The mechanisms underlying this spatial variation in thermal limits will inform species responses to changing temperatures but have rarely been studied (but see^17^). Shifts in gene expression likely contribute to physiological responses that underly range-wide variation in thermal tolerances, responses that may also be shaped by natural selection and ultimately limit species distributions^5,10,18^.

Mountain ranges provide a unique opportunity to investigate how organisms respond to thermal extremes, as temperature declines both with increasing latitude and over shorter distances with increasing elevation^13,19^. Therefore, montane organisms can face similar abiotic extremes at multiple spatial scales, and sampling across latitude and altitude can provide evidence for climate adaptation by separating the effects of space and environment across a species range^10,13^. The physiology of adaptation to variation in thermal stress with altitude or latitude has been studied both within^18,20–22^ and between^12,23,24^ species. Studies employing populations from both spatial dimensions remain rare but may be especially likely to reveal how selection shapes variation in thermal tolerance and underlying molecular mechanisms.

Bumble bees (Hymenoptera: Apidae: *Bombus*) are large-bodied, eusocial bees that are ecologically and economically important pollinators and common in mountainous regions of the Northern Hemisphere^25^. Bumble bees are facultatively endothermic, capable of elevating body temperatures when necessary (e.g., for foraging, incubating brood, etc.), but able to conserve energy by allowing body temperature to track ambient temperatures. This ability and related morphological, behavioral, and physiological adaptations enable bumble bee activity at low ambient temperatures^26^, and facilitate their success in cold environments^27,28^. We thus expect physiological responses of bumble bees to cold are likely to be especially important for shaping their past and present distributions^29^. However, recent bumble bee declines^30^ may in part stem from rising temperatures^31^, suggesting that physiological responses to heat may also determine persistence of bumble bee populations and species. Critically, as responses to thermal extremes may vary among populations particularly for widely distributed species^18,32^, such population variation in thermal tolerance could profoundly alter predictions of the impacts of changing climates on bumble bees.

**Figure 1:**
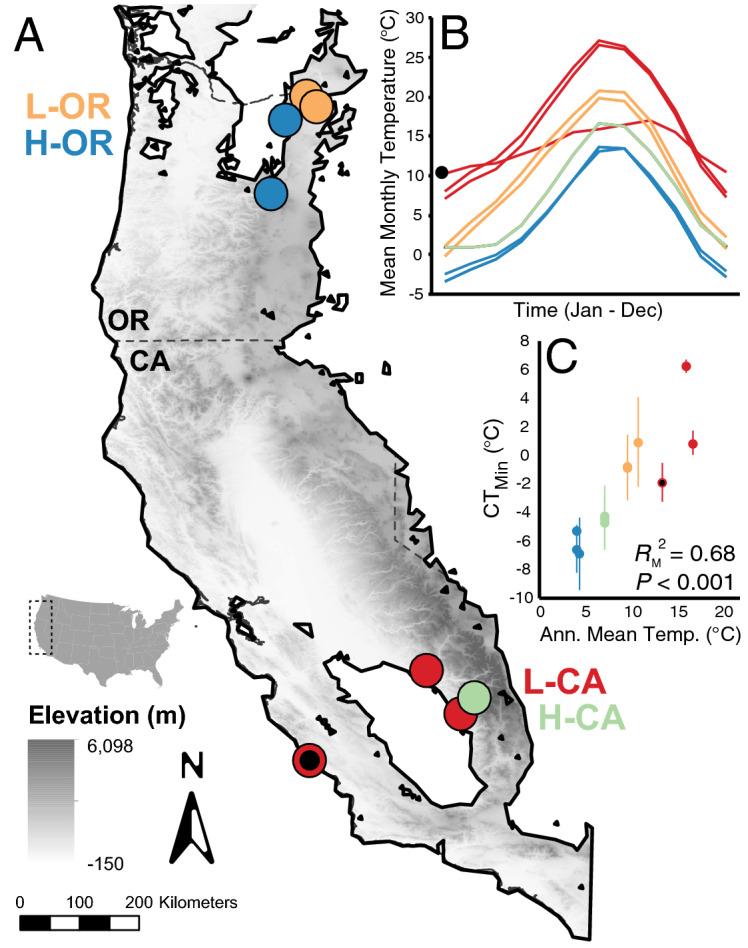
Collection location and physiological tests. (**A**) Queens for laboratory colony rearing were collected from sites near the spatial and bioclimatic extremes of the *Bombus vosnesenskii* range from relatively low and high elevations in southern CA and northern OR (Table S1). The map was created in ESRI ArcMap 10.8, with state outlines and digital elevation data obtained from https://www.diva-gis.org/Data. The *B. vosnesenskii* range outline was taken from the species distribution model published perviously^3,33^. (**B**) Mean monthly temperatures over the course of a year for queen collecting sites. (**C**) The CT_MIN_ temperature of laboratory reared workers is strongly correlated with the annual mean temperature of collection sites of the founding queens. The coastal L-CA colony discussed in Results is indicated by a black dot in each panel.

To investigate intraspecific variation in functional responses to thermal stress, we measured CTLs and gene expression among *Bombus vosnesenskii* sourced from populations from southern California (CA) and northern Oregon (OR), USA, near the extremes of the species range (Fig. 1). *B. vosnesenskii* occurs across broad altitudinal ranges throughout its distribution, and is characterized by weak genetic differentiation (genome-wide *F*_ST_ = 0.003), facilitating identification of strong local effects with little confounding influence from population structure^3,33^. We employed a common-garden style approach with laboratory colonies reared from queens collected at high and low elevations at both high and low latitudes to separate species-wide responses to thermal stress from population-specific differences that may reflect local adaptation. We measured CT_MAX_ and CT_MIN_ in laboratory-reared *B. vosnesenskii* workers and employed transcriptomics (RNAseq) to test how gene expression responses to extreme thermal stress correlate with population-specific thermal tolerance and environmental conditions at the population of origin.

We use this system to address a series of related hypotheses. First, we hypothesize that local adaptation will match critical thermal limits to local environmental conditions^7,8,34–36^, predicting that bees from warmer and colder sites have higher and lower CTLs, respectively. Selection may not be the same at hot and cold extreme temperatures^14,23,37^, however, which could also produce contrasting population-environment correlations at CT_MAX_ and CT_MIN_. Alternatively, if variation in thermal tolerance is largely shaped by flexible or plastic individual responses, then thermal limits should be independent of region of origin^38,39^. Parallel hypotheses can be examined for gene expression. If intraspecific variation in gene expression contributes to population level differences in thermal tolerance, we hypothesize that differential gene expression will vary among populations under thermal stress treatments. If responses to temperature are largely plastic, we expect that treatment (CT_MAX_ vs CT_MIN_) will have the largest effects on expression, with no effect of environmental temperature at the region of origin. Finally, we aim to identify key molecular processes involved in the bumble bee temperature stress response, and especially in population-specific variation in this response that may provide possible targets of local adaptation.

## Results

### Bumble bee cold tolerance varies regionally, tracking local temperatures

Laboratory colonies of *Bombus vosnesenskii* were established from wild queens collected from four regions: low-elevation CA (L-CA), high-elevation CA (H-CA), low-elevation OR (L-OR), and high-elevation OR (H-OR) (Fig. 1A, “SI Appendix”, Table S1), with each region representing a distinct thermal environment, as highlighted by mean monthly temperature over the course of an average year (Fig. 1B). CT_MIN_ of laboratory-reared *B. vosnesenskii* workers varied significantly with region of origin (ANOVA, F_3,8_ = 8.6, *P* = 0.007; Table S2; Dataset S1), with bees from colder regions having reduced CT_MIN_ (Fig. 1C). Across colonies, CT_MIN_ increased significantly with Annual Mean Temperature (AMT) at queen collection localities, with bees from H-OR colonies tolerating temperatures as low as − 7 °C, 12 °C lower than L-CA bees (linear mixed model^40^, *t*_10_ = 5.9, *P* < 0.001; Fig. 1C; Table S3). CT_MAX_ averaged 51.6 °C (4.4 °C SD) across colonies (N = 13) and did not vary significantly among regions (ANOVA, F_3,8_ = 3.01, *P* = 0.1, Table S2) or show a significant association with local temperature (*t*_10_ = 5.9, *P* = 0.2; Table S3, Dataset S1).

### Region of origin and temperature both effect expression patterns

**Figure 2:**
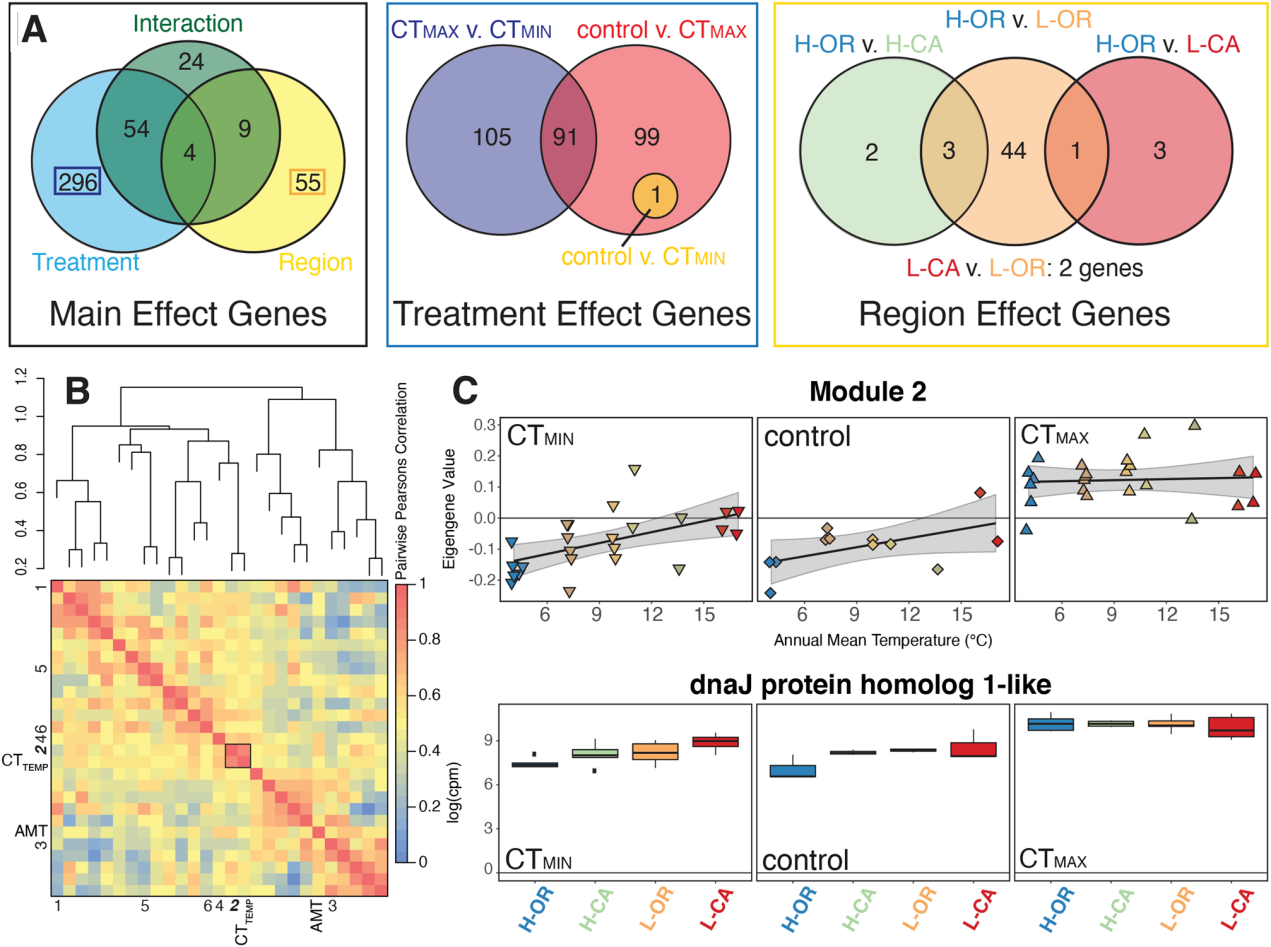
Summary of differential gene expression analysis patterns. (**A**) Venn diagrams summarizing the total number of genes demonstrating significant FDR-corrected (5%) effects, including (left) genes with treatment, region, or interaction effects, (center) genes differentially expressed among CT_MAX_, CT_MIN_, and control treatments for the 296 genes exhibiting treatment effects alone, and (right) 55 genes with region effects alone. See Dataset S2G for details. (**B**) Network analysis of co-expression with WGCNA grouped genes into 26 modules, six of which (labeled 1–6 on heat map edges) demonstrated a significant relationship (likelihood ratio test, *P *< 0.05) with critical thermal temperature (CT_TEMP_), Annual Mean Temperature (AMT), or some combination of the two. The strongest relationship was observed for Module 2 with CT_TEMP_ (black box; Dataset S3). (**C**) The eigengene value (expression analog) of module 2 genes (top) and expression of the module 2 hub gene, dnaJ protein homolog 1-like (bottom) as functions of Annual Mean Temperature for each treatment.

We performed RNAseq on bees from the CT_MIN_ and CT_MAX_ experiments plus untreated controls, and first explored the data by determining best fit general linear models for expression patterns for samples grouped by geographic region (L-CA, H-CA, L-OR, H-OR) or treatment (CT_MIN_, CT_MAX_, control) with the Akaike Information Criterion (AIC)^41^. This preliminary analysis suggested that both experimental treatment and region of origin altered expression patterns, with 46% of genes demonstrating a best-fit model incorporating the experimental temperature at CTL (CT_TEMP_), AMT, or some combination (Table S4, Dataset S2). We then tested specific hypotheses for differential expression at individual genes by specifying contrasts using *edgeR*^42^ and *limma*^43^ (blocking by colony). We identified significant differential expression in at least one treatment, region, or treatment-by-region contrast in 442 genes (Benjamini–Hochberg^44^ FDR ≤ 0.05, Dataset S2). Most of these significantly differentially expressed genes (N = 354) had a significant treatment effect, with 296 genes uniquely differentially expressed between all three pairwise treatment contrasts (Fig. 2A; Dataset S2G). Sixty-eight genes demonstrated differential expression due to region of origin, with 55 genes unique to regional comparisons (Fig. 2A; Dataset S2G). Region-specific effects were primarily driven by unique gene expression of H-OR bees following thermal stress (Fig. 2A).

### Bumble bees show a strong treatment effect on gene expression driven by CT_MAX_

**Figure 3:**
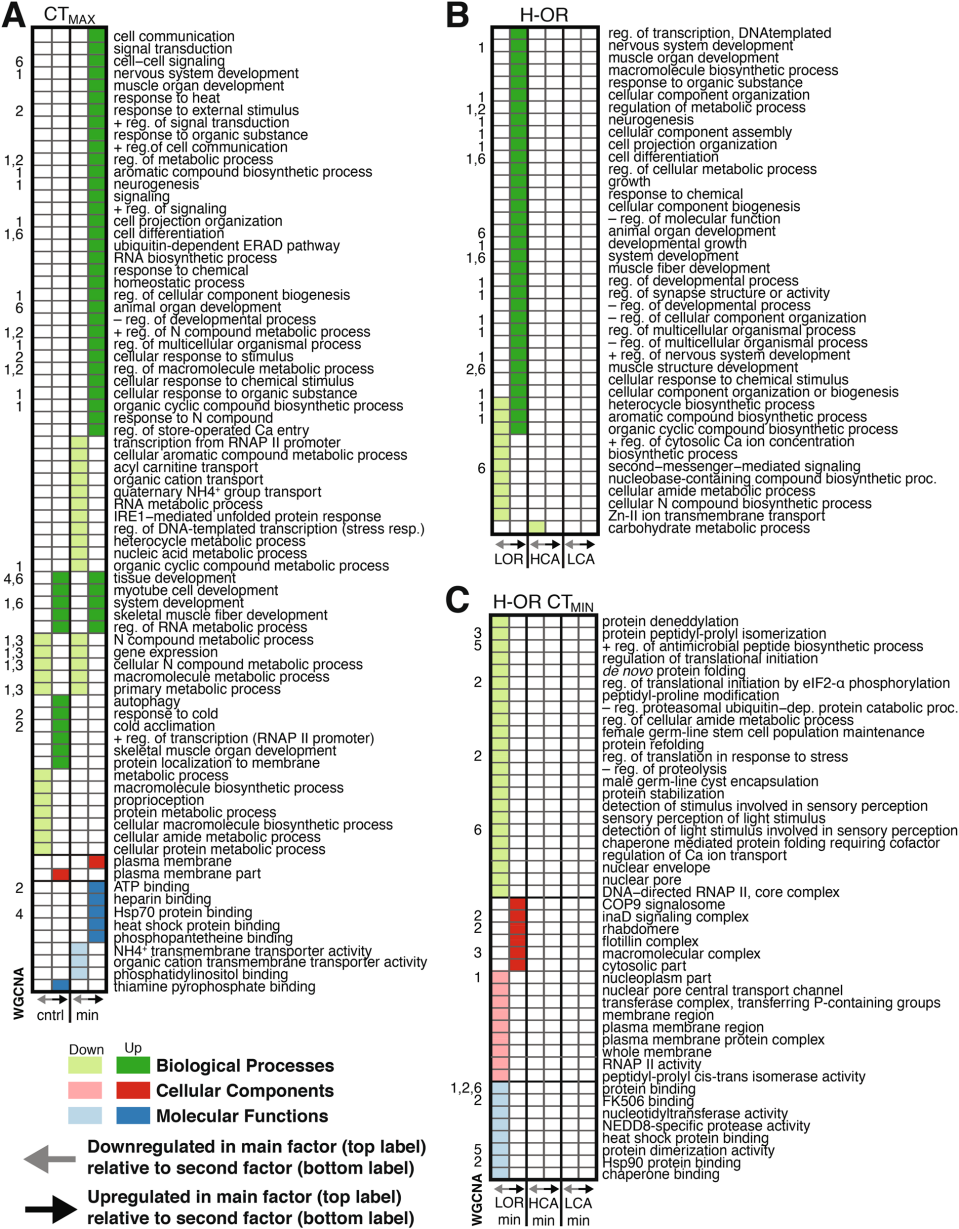
Gene Ontology enrichment analysis of edgeR/limma and WCGNA results. Significantly enriched gene ontology terms identified in edgeR/limma are listed on the rows for different contrasts, with information on the WCGNA modules (1–6) that are also enriched for that term. Each panel has a different main factor for the contrast (top label), with GO enrichment in genes with decreased or increased expression in the main factor relative to the contrast factor (bottom label). (**A**) Treatment comparisons (control and CT_MIN_) against CT_MAX_ treatment, presenting full GO analysis results (Dataset S2) reduced with ReViGO and further filtered to include common terms from other thermal stress studies for the purposes of presentation clarity. (**B**) Region comparisons (L-CA, H-CA, and L-OR) against H-OR bees, with terms reduced as in (**A**). (**C**) Regional CT_MIN_ comparisons (L-CA_MIN_, H-CA_MIN_, and L-OR_MIN_) against H-OR_MIN_ bees. GO terms were summarized with ReViGO but did not require further filtering for presentation clarity. Complete sets of GO terms for all edgeR/Limma and WCGNA results are provided in Datasets S1 and S2, respectively.

Bees warmed to CT_MAX_ showed the clearest differential expression relative both to control bees and to bees cooled to CT_MIN_ (Fig. 2A). Of the 296 genes unique to the treatment contrasts (i.e. not also associated with region or treatment-by-region interactions; Fig. 2A), 91 were differentially expressed between CT_MAX_ and both CT_MIN_ and control. Genes with increased expression in CT_MAX_ compared to other treatments were significantly enriched in GO terms including cellular response to temperature stimuli, heat shock protein binding, signaling, and plasma membrane structure (Fig. 3A, Dataset S2). Genes with decreased relative expression in CT_MAX_ covered a broader range of GO terms, including RNA polymerase II (RNAP II) transcription and gene expression, metabolic pathways and processes, and transmembrane transporter activity (Fig. 3A, Dataset S2). Comparisons between CT_MAX_ and CT_MIN_ revealed altered expression of genes in several relevant GO categories that did not overlap with comparisons between CT_MAX_ and control, including neurogenesis, nervous system and muscle development, response to heat, and transmembrane transport activity (Dataset S2). In contrast to these strong expression differences in response to CT_MAX_, only one gene (uncharacterized, LOC105681062) was differentially expressed between control and CT_MIN_ treatment bees across colonies (Dataset S2G) and less stringent FDR correction (10%) identified only three additional genes (Fig. S1), suggesting a subtler species-wide (i.e. not considering population effects) genomic response to cooling.

### Bees from the coldest sites have pronounced region-specific gene expression

Bees from the coldest sites (H-OR) showed the broadest thermal tolerance range, driven by strongly reduced CT_MIN_ (Fig. 1C). Of the 55 genes that were differentially expressed solely due to population of origin (Fig. 2), 53 involved pairwise comparisons with H-OR (Dataset S2). These included increased expression in H-OR of genes related to DNA-templated transcription regulation and muscle and synapse development and activity, as well as reduced expression of genes related to transmembrane transport (calcium and zinc) and biosynthetic pathways such as ribosome biogenesis (Fig. 3B).

Although 91 genes were significantly differentially expressed in at least one region-by-treatment contrast, most of these were also significant in at least one main effect contrast (Fig. 2A). To clarify discussion and address our main hypotheses about regional local adaptation under thermal stress, we focus primarily on contrasts between regions for each treatment. Control bees did not show differential gene expression related to region of origin. However, bees from different regions differed in their responses to being cooled to CT_MIN_ or heated to CT_MAX_. Only five genes had regional effects under CT_MAX_, all of which were only differentially expressed between H-OR vs. L-OR. The greatest number of genes (N = 14) exhibiting within-treatment population of origin effects were in CT_MIN_ treatment bees, all involving contrasts with H-OR_MIN_ (14 genes with L-OR_MIN_, two with L-CA_MIN_, and one with H-CA_MIN_). Most (13 of 14) were downregulated in H-OR_MIN_ relative to L-OR_MIN_ and are involved in GO processes such as protein folding and stabilization, heat shock (and Hsp90) protein and chaperone binding, nuclear pore transport, and translation regulation. Upregulated GO terms for H-OR_MIN_ related to cell membrane composition/flotillins.

Notably, relatively few genes and GO terms were detected as significant between H-OR and L-CA bees, despite the large differences in CT_MIN_ (Figs. 2A, 3B,C). One possible explanation is that one L-CA colony came from a coastal locality with unusually low annual temperature variation and correspondingly low CT_MIN_ relative to other L-CA bees (Fig. 1B,C). Exclusion of this colony from analyses would have reduced statistical power for categorical group comparisons, and so we instead opted to take into account colony-specific environmental and physiological information through WCGNA gene expression module analysis.

### Linking region of origin, physiology, and gene expression with gene expression modules

To test how gene expression is shaped by both source environmental conditions and physiological responses of individual bees under thermal tolerance treatments, we employed network analysis with *WGCNA*^45^ to reduce the complex gene-by-gene patterns into covarying expression modules. Genes clustered into 26 modules, 13 of which correlated significantly with predictors of interest in initial analyses (Dataset S3). The most significant association in initial univariate correlation analysis was between Module 2 and CT_TEMP_ (*R*_*CT*_ = 0.74, *P* = 1.8 × 10^–11^). Likelihood regression analysis of the eigengene values (i.e. expression) of environmentally correlated modules identified six modules that were significantly best fit by a linear model that included AMT, CT_TEMP_, or some combination of the two, and we focus the remainder of our discussion on these (Dataset S3).

Module 2 produced the strongest relationship between expression and explanatory variables of all modules [best fit by the model *eigengene* ~ *1* + *AMT* + *CT*_*TEMP*_ + (*1|Colony*) (LRT: *P* = 2.53 × 10^–12^)] and demonstrated the greatest overlap with the *edgeR*/*limma*^42,43^ gene-by-gene analyses (85 of 111 genes). The major effect on Module 2 expression was CT_TEMP_, but region of origin (AMT) effects were clearly apparent in control and CT_MIN_ treatments, with patterns of expression at CT_MIN_ and CT_MAX_ that mirror the physiology data (Fig. 2C). Module 2 was enriched in genes relating to response to stress and stimulus (including cold), transcription regulation activity (particularly gene silencing), and various components of muscle, and contained many of the major significant genes that were differentially expressed among treatments (e.g., heat shock genes) (Dataset S3). The hub-gene was LOC100743914 (Dataset S3), a *dnaJ* homolog and putative small heat shock protein, which was differentially expressed in 16 gene-by-gene *edgeR* contrasts (Dataset S2).

Five other modules were best fit by significant non-null models (0.020 ≤ *P* ≤ 0.049). Module 1 was best fit by a full factorial interaction model [*eigengene* ~ *1* + *AMT* + *CT*_*TEMP*_ + AMT * CT_*TEMP*_ + (*1|Colony*)], and genes in this module (Datasets S1 and S2) were involved in neurogenesis, metabolism, DNA transcription, regulation of growth, and general cell cycle processes (Fig. 3; Dataset S3). Two of the remaining modules were best fit by CT_TEMP_ only models, with highest eigengene expression values for Module 3 and Module 4 genes observed in CT_MIN_ and CT_MAX_ bees respectively. GO terms in Modules 3 and 4 supports results from other analyses, showing increased expression in CT_MIN_ bees related to various catabolic and metabolic processes, gene expression, and transmembrane transporter activity (Fig. 3). Modules 5 and 6 were best fit by AMT only models, with Module 6 in particular containing genes with GO terms related to muscle development, cellular differentiation, protein binding, and cell-to-cell signaling (Fig. 3, Dataset S3C).

## Discussion

This study demonstrates that variation in the local abiotic niche may drive intraspecific variation in physiology and associated molecular responses when bumble bees are exposed to thermal stress, and that population-dependent responses differ with heat versus cold. As predicted, laboratory-reared *Bombus vosnesenskii* worker bees from different geographic regions demonstrate strong population-level differences in cold tolerance (CT_MIN_) and gene expression responses at low temperatures. The correlation between CT_MIN_ and source locality AMT, and the identification of co-expressed gene sets (e.g., Module 2) with parallel responses to AMT and CT_TEMP_, suggest the potential for local adaptation in physiological and gene expression responses. In contrast, although the largest gene expression responses are produced by warming to upper thermal limits, gene expression under heat stress is conserved across populations, which mirrors a lack of interpopulation variation in CT_MAX_ itself. Although direct evidence for selection on CTLs and gene expression will require further work, these data suggest that if local environment shapes thermal tolerance of bumble bees, it may be responses to low temperatures that are most malleable, and that such variation may be in part associated with gene expression. Such results are consistent with the importance of cold adaptation for the evolution of *Bombus* diversity above the species level^27,28^, while also indicating potential constraints for adaptation to increasing temperatures in the warmest parts of species ranges^31,46^.

The importance of temperature extremes as drivers of thermal tolerance evolution is well-known^14^, and several studies indicate that physiological cold tolerance thresholds in terrestrial ectotherms can change along spatial-environmental gradients while upper thresholds are constant^14,23,24,47,48^. Our data extend these observations to facultative endotherms and show complementary patterns for associated molecular processes. A possible explanation for the lack of variation in gene expression and CT_MAX_ is that bumble bees experience high thoracic temperatures during flight regardless of ambient conditions^26^, and have therefore evolved to deal with uniformly high body temperatures. Bumble bees also rely on behavioral thermoregulation to mediate stress at high ambient temperatures (e.g., limiting foraging activity)^49–51^. The heat shock response is clearly robust based on gene expression data, largely relating to processes including unfolded protein binding, signaling, regulation of transcription and gene expression, metabolic pathways and processes, and transmembrane transporter activity^52–54^. However, limited variation in the point at which this response is initiated across populations indicates there may be physiological or molecular constraints on the evolution of heat tolerance^55,56^.

Variation among source populations in gene expression at CT_MIN_ suggests several mechanisms underlying the physiological response to cold stress that may be shaped by local adaptation. The onset of chill coma at CT_MIN_ produces spasms that are likely symptomatic of failure at the neuromuscular junction or locally in the muscle^57^, and differentially expressed genes involved in neurogenesis and muscle structure and function are detected in both treatment and region analyses (Dataset S3C). Failure of the neuromuscular junction can involve changes in ion transport channels that regulate membrane potential^57^. Calcium regulation is especially important for the cold tolerance response^58^ and, for example, H-OR bees demonstrated decreased expression in a homolog of *calumenin* (LOC100743248), a gene known to play a role in thermal stress tolerance^54,59^. Differences in membrane composition, rigidity, and permeability are also important for the cold shock response, and increased expression of *flotillin-1* (LOC100740847) in H-OR bees^60^ reflects the potential importance of cell membrane adaptations among *B. vosnesenskii* populations^58,60–62^.

One class of genes, the small heat shock proteins (sHSPs), was involved in responses to both high temperatures and population-specific responses to cold. Geographic variation in expression of HSPs is known to influence adaptation for thermal tolerance across species ranges^8,18,22,63,64^. The importance of sHSPs in *B. vosnesenskii* was clearest in the WGCNA module analysis; for example, the hub gene of Module 2 (Fig. 2) is the sHSP dnaJ, and this module contains numerous HSPs that were overrepresented in various treatment and region contrasts (e.g., LOC100742443-protein lethal(2)essential for life-like is differentially expressed in 14 different contrasts including control vs. CT_MAX_, CT_MAX_ vs. CT_MIN_, and H-OR vs. L-OR). The consistent regional expression patterns of *BAG domain-containing protein Samui-like* (another Module 2 gene), especially involving H-OR bees, is also of interest because this gene interacts with HSPs in the cold stress response and maintenance of muscle membrane potential, and has previously been detected as a key player in cold stress for other bees^65,66^. Intraspecific variation in both constitutive and inducible expression of heat shock and related proteins is thus one mechanism that may underly adaptation to hot and cold thermal extremes in *Bombus* populations.

Beyond the extreme temperature responses revealed here, there are several avenues for future work. First, there is evidence that bumble bees can show signs of heat stress at ambient temperatures as low as 36 °C^67^, significantly lower than CT_MAX_ in *B. vosnesenskii*. Our assays reflect physiological limitations^68^ at extreme temperatures; but behavioral and ecological responses of bees to less extreme temperatures may also vary among populations and should be investigated. Second, our results suggest that cold tolerance may be more responsive to local selective pressures than heat tolerance, but worker bees are typically active during periods where ambient temperatures as low as the observed CT_MIN_ values are uncommon. Worker CT_MIN_ temperatures are closer to those that might be experienced by queens during overwintering or nest founding, which are likely strong targets of selection^69^. Our results show some overlap with differentially expressed genes observed in diapausing/post-diapausing *B. terrestris* queens and some *Megachile*^66,70^, and future research on old stress in queens and links to worker physiology would be particularly informative for understanding true targets of selection for cold tolerance. A related caveat is that while experimental workers were reared in the lab and never experienced field conditions that would alter their physiology via plasticity or acclimation, queens did overwinter in the field, which could contribute to maternal effects relating to region of origin^71,72^. Such effects might be avoided by rearing second-generation colonies, but this proved unsuccessful for *B. vosnesenskii* and is a technical challenge that must be overcome. Third, we cannot exclude the possibility that the regional correlation between CT_MIN_ and differential gene expression may reflect different extremes of cold stress exposure, owing to the different temperatures at which populations enter chill coma. Unfortunately, because of such regional differences in CT_MIN_ it is impossible to evaluate gene expression patterns in all bees for the same critical physiological threshold. Based on our results here, future studies might thus be designed to address related questions by, for example, comparing gene expression among populations at the same temperatures through time during the cooling process. Finally, we studied thoracic muscle given its importance in bumble bee thermoregulation and flight^26,50,73^, however, head and abdominal tissues may show different levels of sensitivity to heat or cold exposure^74^. Ultimately, additional experiments will be necessary for untangling contributions to cold tolerance from genetic adaptation, plasticity, maternal effects, and roles of different tissues.

In conclusion, we have demonstrated that bumble bees exhibit conservation in physiological upper thermal tolerances and gene expression, and interpopulation variation in lower thermal tolerances and gene expression that match conditions in their local niche. Our results provide one possible mechanism that could link global climate change to bumble bee range contractions, which appears to result from erosion in southern and low-lying regions combined with failure to colonize north of existing range margins^31^. Populations in warmer regions may experience environmental temperatures which exceed their inflexible upper thermal limits, while poleward or upslope range expansions could be limited if cold extremes at available sites exceed CT_MIN_ of dispersers. These conclusions have implications for bumble bee conservation, suggesting that managers may need to consider future changes in temperature extremes as well as means^24,75^, and should also include evaluation of population-specific phenotypes and genomic variation^76^. Additional work to characterize physiology and functional genomics at the spatial and abiotic extremes of *Bombus* species ranges must remain a priority if we are to understand the potential for adaptation to changing temperatures.

## Methods

### Queen collection and thermal physiology experiments

Queens were transported from collecting sites to the USDA Pollinating Insect Research Unit (PIRU) (Logan, UT USA) for colony initiation (“SI Methods”). Colonies with > 20 workers (N = 13, 3–4 per region) were transported to the University of Wyoming (Laramie, WY USA) for experiments, where they were maintained at 25 °C and 12:12 h L:D cycles. All experiments used workers that developed under these common laboratory conditions from wild caught queens. While conditions in lab are different from those experienced in the field for all populations, these conditions are standardized across colonies. Experiments using workers raised in the lab prevents individual plasticity arising from field exposure, but queens are wild, so maternal effects could be present. Unfortunately, it was not possible to raise a sufficient number of second-generation colonies from lab-reared queens. Environmental data for each collection site was extracted from WorldClim v2 (0.5 min resolution)^77^.

Physiological tests were conducted as part of a larger study of *Bombus* thermal physiology using validated techniques for measuring critical thermal limits^68^. Workers were arbitrarily selected, placed in individual 2-dram clear glass vials, and randomly assigned to control (room temperature, 22 °C), upper (CT_MAX_) or lower (CT_MIN_) temperature treatments. Vials and a thermocouple were placed in machined wells in a solid aluminum block attached to an insulated thermoelectric plate. For CT_MIN_ and CT_MAX_, bees were held at 25 °C for 15 min before decreasing or increasing the temperature at 0.25 °C min^−1^, respectively^78–80^. At CT_MIN_, bees exhibit characteristic signs of “chill coma”, while CT_MAX_ is indicated by uncontrollable muscle spasms^68^. Upon reaching thermal limits, bees were immediately trisected between body sections using a sterile scalpel, placed in RNAlater (Qiagen), and stored at − 80 °C. We used repeated measures ANOVA and linear mixed effects models^40^ (degrees of freedom and *P* values determined with *lmerTest*^81^) to test for regional differences in CT_MIN_ and CT_MAX_ (“SI Methods”; Dataset S1), as well as correlation with various BioClim^77^ variables characterizing the environmental variation in thermal conditions, including Annual Mean Temperature, Minimum Temperature of the Coldest Month, Maximum Temperature of the Warmest Month, and Mean Diurnal Range (Fig. S2; Table S3). Although CTLs can be shaped by extremes and variability of local climate, we focus on Annual Mean Temperature as a metric to characterize the average climate at each collecting site because it is strongly correlated with the other environmental variables of interest and CTL patterns were similar across multiple variables (Fig. S2).

### RNA extraction, sequencing, and bioinformatics

Samples for RNAseq were arbitrarily ordered to avoid batch effects. Whole RNA was extracted from thoracic muscle (E.Z.N.A. Total RNA Kit II; Omega Bio-tek, Inc.) because of the role of musculature for thermogenesis and thermal tolerance^50,73,82^ and the importance of homogenous tissues in RNAseq^83^. We analyzed one control bee and two bees from each CT treatment per colony, except in one case (N = 60 bees from 13 colonies; SI Table S1).

Each bee was sequenced via poly-A amplified directional 50 bp paired-end RNAseq to ~ 20 M reads (± 2.1 M SE) on a HiSeq 2500 (Illumina, Inc) by HudsonAlpha Institute for Biotechnology (Huntsville, AL) (data available on the Sequence Read Archive SAMN13234508-13234603). Reads were processed using TrimGalore v0.4.2^84,85^ and aligned to the *Bombus impatiens* genome v2.0 (BIMP2.0^86,87^) with TopHat v2.1.1^88^ (stranded, maximum three mismatches permitted, intron length distance 20–50,000 bp, mate inner distance 400 ± 30 bp). Gene ontologies for BIMP2.0 were assigned using a reciprocal best hit approach^89^ from the *Drosophila melanogaster* genome. Using NCBI BLAST + 2.9.0^90^, blast databases were created from the protein sequences of both BIMP2.0 and *D. melanogaster* assembly Release 6 plus ISO1 MT. *Bombus impatiens* genes which were identified as a single *D. melanogaster* gene’s best scoring hit in reciprocal subject-query *blastP* analyses (e-value threshold ≤ 10^–5^) were assigned the GO term(s) associated with its fly ortholog in Ensembl 2018^91^ using biomartr v0.9.0^92^. In total, 7088 genes had *D. melanogaster* orthologs, and 6645 of these were assigned GO terms.

### Gene expression and gene ontology enrichment analyses

RNAseq analyses used *edgeR* v3.22.3^41,42^ and *limma* v3.36.2^43^. Raw counts were transformed to counts per million (CPM), retaining genes with ≥ 1 CPM in ≥ 2 samples. Analyses were blocked by colony with *limma duplicate*Correlation. Analysis was then conducted in three stages: (1) model-based sub-setting using AIC; (2) individual gene-by-gene differential expression analyses; (3) gene co-expression module analyses.

We first used *limma select*Model to determine a best fit general linear model for each gene from alternative nested models (Region, Treatment, Region + Treatment, or Full model with an interaction) with both the default results, as well as the model with the smallest number of parameters (“simplest model”) with an AIC score within 2 of the minimum AIC^41^ (Table S4, Dataset S2A).

Second, we performed individual gene-by-gene analyses with a contrasts approach in *edgeR* to test for region, treatment, and region-by-treatment effects on gene expression. Significance was assessed at a False Discovery Rate (FDR) ≤ 0.05, using Benjamini–Hochberg adjusted P-values^44^ of all genes (N = 9328) and contrasts on a per-factor basis (e.g. three pairwise contrasts between treatments; six pairwise contrasts between region levels; 66 region-by-treatment pairwise contrasts for the interaction term).

Finally, to identify clusters of genes with co-expression patterns (modules) and correlate expression with individual-specific physiological results and environmental data (versus regional groupings of queen collection sites used above), data were assigned to expression modules using weighted gene co-expression network analysis in WGCNA v1.64.1^45^ following best practices and previous studies^18,93^. As predictors, we used Annual Mean Temperature (AMT) at queen collection sites to capture effects of local environment, and CT_TEMP_ (i.e. experimental CT_MIN_ or CT_MAX_, controls = 22 °C) at which each bee was sampled to capture treatment effects. We retained modules with significant responses to AMT and/or CT_TEMP_ (*P *≤ 0.05 from function *corPvalueStudent*). For each filtered module, we used linear mixed-effects models (*lmer*^40^) to identify a best fit model (colony as a random effect; AMT and CT_TEMP_ as fixed effects, including additive and interaction terms) for gene expression (WGCNA eigengene value) (“SI Methods”).

Most genes were expressed in two or more samples (80%; 9328/11,600), including most GO-annotated genes (95%; 6327/6645). GO enrichment analyses were performed with topGO v2.30.0^94^ using classic Fisher’s Exact Test with default settings^95^ for biological process (BP), molecular function (MF), and cellular component (CC). We consider enrichment significant at *P *≤ 0.05, as we were primarily interested in examining functional information for genes that were already stringently selected following conservative FDR correction, and GO enrichment *P* values do not conform to distributions typically required for FDR correction^96^. For ease of visualizing GO terms in presented results, GO lists were simplified using ReViGO^97^ (allowed similarity = small), and then filtered for terms (with parent and child terms) previously identified as enriched in thermal stress studies in other organisms^58,98–100^. However, complete unfiltered GO results can be found in Datasets S1E and S2C, and intermediate datasets and R code can be found on DRYAD (10.5061/dryad.tmpg4f4wx).

## Supplementary information

Supplementary Information.

Supplementary Dataset 1.

Supplementary Dataset 2.

Supplementary Dataset 3.
