# Supplementary material for: Biogeographic parallels in thermal tolerance and gene expression variation under temperature stress in a widespread bumble bee
Source: Sci Rep. 2020 Oct 13;10:17063. doi: 10.1038/s41598-020-73391-8 (PMC7553916; doi:10.1038/s41598-020-73391-8)
Supplement: Supplementary file 1 — Supplementary Information. [file 41598_2020_73391_MOESM1_ESM.pdf]

Supplementary Information for

Biogeographic parallels in thermal tolerance and gene expression variation under temperature stress in a widespread bumble bee

Meaghan L. Pimsler[1*], Kennan J. Oyen[2,3], James D. Herndon[4], Jason M. Jackson[1], James P. Strange[4,5], Michael E. Dillon[2], Jeffrey D. Lozier[1*]

[1] Department of Biological Sciences, The University of Alabama, Tuscaloosa, Alabama 35487, USA

[2] Department of Zoology and Physiology and Program in Ecology, University of Wyoming, Laramie, Wyoming 82071, USA

[3] Department of Biological Sciences, University of Cincinnati, Cincinnati, Ohio, 45221, USA

[4] USDA-ARS Pollinating Insects Research Unit, Utah State University, Logan, Utah 84322, USA

[5] Department of Entomology, The Ohio State University, Columbus, Ohio 44691, USA

[*]To whom correspondence should be addressed: Meaghan L. Pimsler
mlpimsler@gmail.com
Jeffrey D. Lozier
jlozier@ua.edu

This PDF file includes:

- Supplementary text
- Fig. S1 to S2
- Tables S1 to S4
- Captions for Datasets S1 through S3
- References for SI citations

Other supplementary materials for this manuscript include the following:

- Datasets S1 to S3

Supplementary Information Text

Methods

Queen Collection and Colony Foundation.

Queen *Bombus vosnesenskii* bees were collected by net from multiple sites between February and July 2016 (Table S1). To increase chances of successful laboratory colony foundation, we focused on queen bees which had recently emerged from hibernation but had not yet started a colony by collecting queen bees visiting flowers. As is typical of many bumble bees, hibernating queen emergence varies seasonally with latitude and elevation[1], and it was not possible to collect queens from all regions in the same month. Queens were collected while foraging or flying using aerial insect nets, assigned a unique identification code, transferred to shipping vials and placed in a cooler for transport to the USDA-ARS Pollinating Insect Research Unit (PIRU) bumble bee rearing facility.

At PIRU, queens produced colonies following a protocol based on procedures by Evans et al.[2], with modifications to maximize *B. vosnesenskii* production. Queens placed in plastic queen initiation boxes (2.25L) (Biobest, Leamington, ON), provided with a 500 mg pollen provision (homogenous paste made of honey bee corbicular loads and sugar solution) dipped in honey bee wax to encourage nesting behavior. Each queen was also provided lab-made nectar solution in a plastic reservoir *ad libitum*. The queen initiation boxes were maintained at 0:24 L:D, 27° ± 1° C and 55-60% relative humidity (RH)[3]. Queens were checked daily for signs of nesting behavior, including wax secretion, honey pot construction, or presence of brood. After five workers had eclosed, the small colony was moved to a 7.75 L plastic hive box (Biobest, Leamington, ON). Additional pollen provisions (without a wax coating) were provided as needed. At 20+ workers, colonies were transported to the University of Wyoming for physiological experiments. During the course of nest initiation through the transport to the University of Wyoming, the queens and colonies were checked daily for presence of disease and pests and managed to maximize colony growth. Propagation, mating, and overwintering of queens for second-generation laboratory reared colonies was attempted but not possible for this species.

Physiology Data Analysis.

We tested for statistical differences between queen collection locations for thermal maxima and minima in two separate pairs of analyses. All data were analyzed in R v3.5.1[4]. First, we tested for broad patterns based on region of origin used a mixed effect linear model approach, defining colony of origin as a random effect, using *lmer* from lme4 v1.1-18-1[5] and lmerTest v.3.1-1[6]. Next, we also tested for a relationship between either CT_{MAX} or CT_{MIN} and specific queen collection location using *lmer* and calculated the R^2 of the relationship using *r.squaredGLMM* from MuMIn v1.42.1[7], against the following WorldClim[8] variables: Annual Mean Temperature, Maximum Temperature of the Warmest Month, Minimum Temperature of the Coldest Month, Mean Diurnal Range, and Isothermality.

Weighted Gene Co-Expression Network Analysis.

To study broad patterns of gene expression, data were analyzed using WGCNA v1.64.1[9] following best practices as outlined in the package tutorials to identify modules of co-expressed genes that correlated with physiological and environmental variables,

similar to previous studies[10,11]. The data were prepared by quantile normalizing the filtered *logCPM* data used in the MDS and gene-by-gene (edgeR/limma[12,13]) analyses. Next, these data were used construct a signed-hybrid network and classify modules using the step-by-step approach using pairwise Pearson correlations and a soft-threshold power of 6, as identified by *pickSoftThreshold*. Next, modules were identified with cutreeDynamic and merged if they demonstrated a correlation >75%. The resultant modules were then compared to environmental and physiological traits of the individual bees using Pearson correlation coefficients. The traits tested were: ColonyID, Latitude, Longitude, Elevation, Region, Treatment, Region by Treatment interaction, Annual Mean Temperature of queen collection location (from WorldClim) and CT_{TEMP} (temperature of the individual bee at time of collection with control bees assigned 25°C). The modules with statistically significant correlation ($P \leq 0.05$, *corPvalueStudent*) to at least one trait variable was retained for further analysis. We then tested for a significant association between the modules and location of origin (using WorldClim Annual Mean Temperature[8] as a proxy) and temperature at time of sacrifice (using CT_{TEMP}). We compared four linear mixed models of decreasing complexity (ColonyID as a random variable) using the eigengene value of each member of the module as the response variable. The model with the best fit to the module members was identified by iteratively comparing nested models using likelihood ratio tests[14] as implemented by lmerTest v3.0-1[6] using a $P \leq 0.05$ of a Kenward-Rogers Approximation as our cutoff[15]. The best model was then compared to the null model to determine goodness of fit. Modules were also evaluated to identify the hub gene (gene with the highest level of connectivity to all other module members; *chooseTopHubInEachModule*), which gene ontology groups were enriched (following methods described above), and level of overlap with gene-by-gene differential expression results.

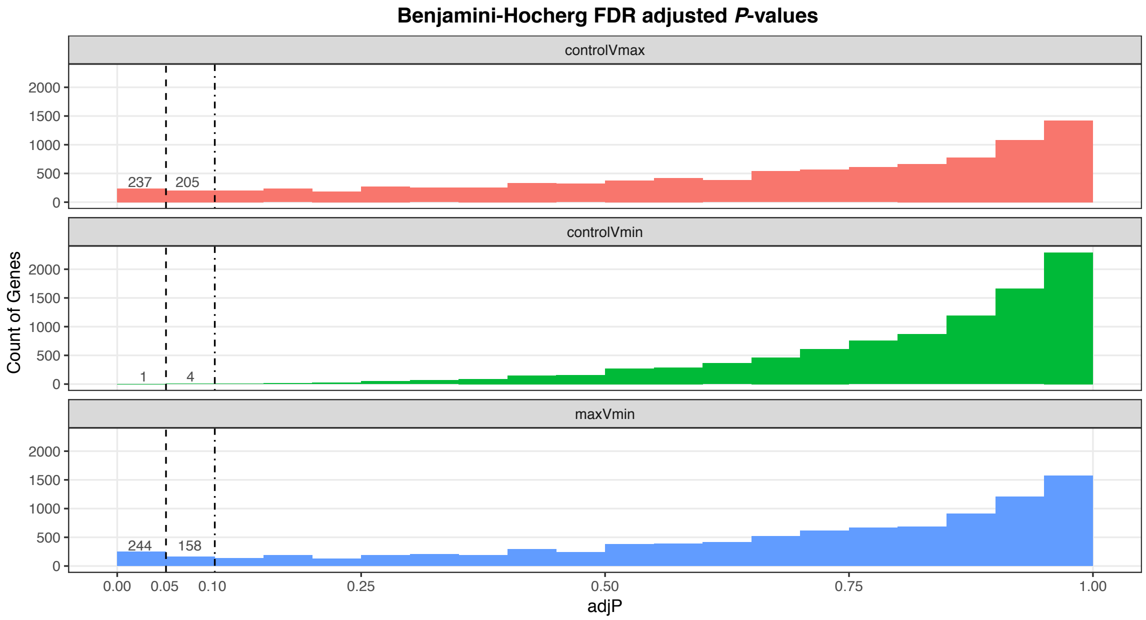

Fig. S1. Histogram of Benjamini-Hochberg false discovery rate corrected p-values for all genes in all three pairwise treatment contrasts. While the distribution of FDR corrected values are similar in distribution for contrasts with CT_{MAX} and both control and CT_{MIN}, very few genes are differentially expressed between control and CT_{MIN} treatment bees at both the $P \leq 0.05$ level (left of the dashed line) and the $P \leq 0.1$ level (left of the dash-dot line).

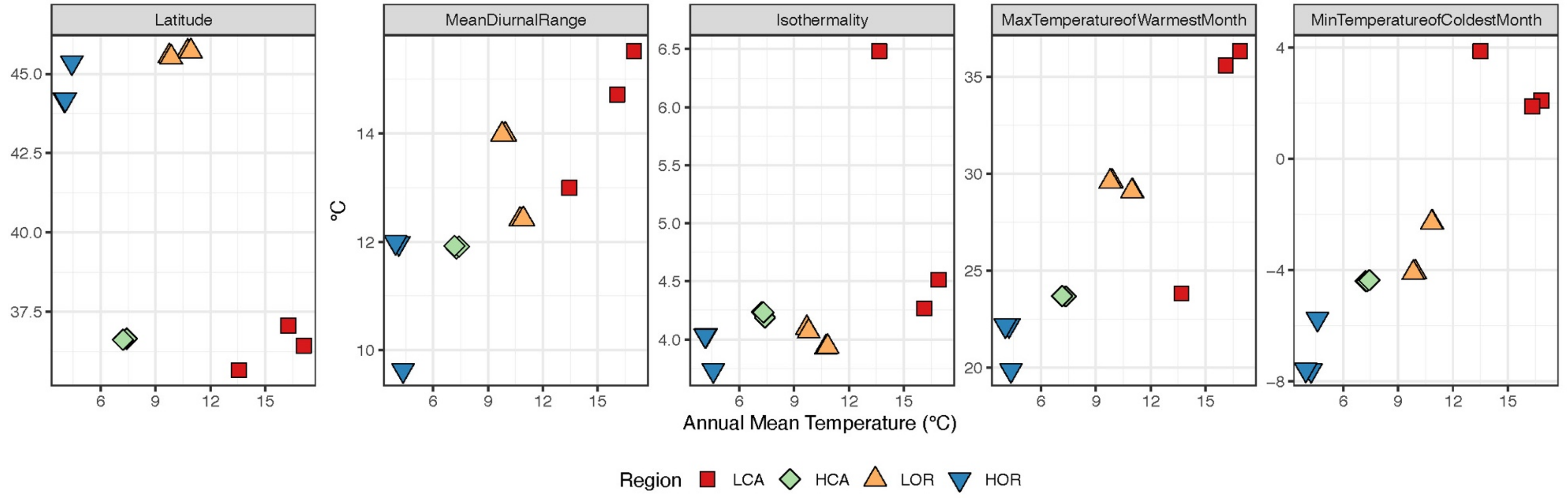

Fig. S2. Annual Mean Temperature is correlated with the other tested BioClim variables, but not with latitude illustrating how our sampling design decouples space and climate.

Table S1. Collection sites for queen bees, with colony IDs matching those of queens

Colony	State	Latitude	Longitude	Elevation (m)	Region
160019	CA	35.68882	-121.28838	7	L-CA
160034	CA	36.39965	-118.99074	224.9	L-CA
160035	CA	37.03608	-119.5266	350.5	L-CA
160095	OR	45.696075	-121.33883	70.1	L-OR
160102	OR	45.696075	-121.33883	70.1	L-OR
160131	OR	45.535039	-121.20785	441.4	L-OR
160137	OR	45.535039	-121.20785	441.4	L-OR
160457	CA	36.63117	-118.80432	2153.7	H-CA
160460	CA	36.63117	-118.80432	2153.7	H-CA
160462	CA	36.63117	-118.80432	2153.7	H-CA
160637	OR	44.22465	-121.8717	1474.6	H-OR
160652	OR	45.3257	-121.65949	1594.7	H-OR
160664	OR	44.22465	-121.8717	1474.6	H-OR

Table S2. CT_{MIN} and CT_{MAX} modeled by region. Region of origin as a categorical variable (repeated measures ANOVA, including colony as a random effect) had a significant effect on CT_{MIN} but not CT_{MAX}.

**ANOVA***						
Treatment	Error strata	Df	SumSq	MeanSq	F	*P*(F)
CT_{MAX}	Error: Colony					
	Region	3	216.9	72.31	3.009	0.095
	Residuals	8	192.2	24.03		
	Error: Within					
	Residuals	12	199.3	16.61		
CT_{MIN}	Error: Colony					
	Region	3	246.4	82.12	8.616	0.00691
	Residuals	8	76.25	9.54		
	Error: Within					
	Residuals	12	35.58	2.965		

* Generalized ANOVA model: CT_{TEMP} ~ Region + Error(ColonyID)

Table S3. CT_{MIN} and CT_{MAX} modeled by five BioClim variables representing aspects of the thermal environment including colony as a random effect using a generalized linear mixed model approach[†]. Four of the tested variables had a significant effect on CT_{MIN} but none had a significant relationship with CT_{MAX}.

		**Annual Mean Temperature**		**Maximum Temperature of the Warmest Month**		**Minimum Temperature of the Coldest Month**		**Mean Diurnal Range**		**Isothermality**	
		(Int.)	AMT	(Int.)	MTWM	(Int.)	MTCM	(Int.)	MDR	(Int.)	Iso
**CT_{MIN}**	Est.	-9.44	0.76	-19.68	0.65	-0.11	0.74	-26.9	1.92	-6.35	0.92
	Stat.	-7.12	5.86	-7.76	6.95	-0.11	3.34	-4.6	4.22	-0.88	0.56
	df	10	10	10	10	10	10	10	10	10	10
	p	***<0.001***	***<0.001***	***<0.001***	***<0.001***	0.917	***0.007***	***0.001***	***0.002***	0.401	0.59
	σ^2	2.97		2.97		2.97		2.97		2.97	
	τ_{00}	2.15 ColonyID		1.28 ColonyID		6.14 ColonyID		4.31 ColonyID		14.16 ColonyID	
	ICC	0.42		0.3		0.67		0.59		0.83	
	N	12 ColonyID		12 ColonyID		12 ColonyID		12 ColonyID		12 ColonyID	
	Obs.	24		24		24		24		24	
	Marg.R^2	0.680		0.732		0.448		0.553		0.024	
	Cond.R^2	0.814		0.813		0.820		0.818		0.831	
**CT_{MAX}**	Est.	54.82	-0.37	59.64	-0.31	50.16	-0.4	60.43	-0.71	51.49	-0.02
	Stat.	19	-1.3	9.48	-1.33	31.79	-1.2	5.72	-0.86	6.3	-0.01
	df	9.95	10.04	10	10.11	10.56	9.99	10.09	10.04	10.43	10.29
	p	***<0.001***	0.222	***<0.001***	0.211	***<0.001***	0.256	***<0.001***	0.409	***<0.001***	0.992
	σ^2	16.78		16.85		16.69		16.55		16.6	
	τ_{00}	8.84 ColonyID		8.61 ColonyID		9.32 ColonyID		10.68 ColonyID		11.94 ColonyID	
	ICC	0.34		0.34		0.36		0.39		0.42	
	N	13 ColonyID		13 ColonyID		13 ColonyID		13 ColonyID		13 ColonyID	
	Obs.	24		24		24		24		24	

Marg.R^2	0.090	0.094	0.079	0.043	0.000
Cond.R^2	0.404	0.400	0.409	0.418	0.418

†: Generalized linear mixed model: $CT_{TEMP} \sim 1 + \text{Variable} + (1|\text{ColonyID})$

Table S4. Numbers of differentially expressed genes (DEG) as a function of thermal treatment and/or region of origin from linear models in limma. The total number of genes assigned to a model are based on either the *selectModel* default results or simplest model within 2 of the absolute lowest AIC for colony blocked data. The numbers and percentages of genes within each best-fitting model that are differentially expressed in at least one region, treatment, or region:treatment contrast in the individual gene analyses (FDR cutoff of ≤0.05) are provided in parentheses. Given that the gene assignment to a linear model necessitates exclusion from other possible models, any individual gene may be significant in contrasts that don't align with its linear model categorization.

Model Name	Model Formula	*selectModel* (DEG)	Simplest Model (DEG)
Total Genes = 9328			
None	~ 1	3654 (0, 0%)	5032 (1, 0%)
Region	~ 0 + Region	2855 (57, 2%)	2581 (66, 3%)
Treatment	~ 0 + Treatment	1393 (201, 14%)	1049 (242, 23%)
Treatment + Region	~ 0 + Treatment + Region	1056 (122, 11%)	494 (101, 20%)
Full	~ 0 + Treatment + Region + Treatment*Region	370 (62, 17%)	172 (32, 19%)

Dataset S1 (separate file)

Results of physiological test of thermal maximums and minimums of *Bombus vosnesenskii* bumble bee workers raised in a common laboratory environment from queens collected from altitudinally and latitudinally distinct populations.

Dataset S2 (separate file)

Results of edgeR/Limma individual gene-by-gene analysis of differential gene expression for thermally stressed *Bombus vosnesenskii* bumble bee workers raised in a common laboratory environment from queens collected from altitudinally and latitudinally distinct populations, including gene ontology results.

Dataset S3 (separate file)

Results of WGCNA analysis of patterns of differential gene expression for thermally stressed *Bombus vosnesenskii* bumble bee workers raised in a common laboratory environment from queens collected from altitudinally and latitudinally distinct populations, including gene ontology results.

References

1. Goulson, D., Hanley, M. E., Darvill, B., Ellis, J. S. & Knight, M. E. Causes of rarity in bumblebees. *Biol. Conserv.* **122**, 1–8 (2005).
2. Evans, E., Burns, I. & Spivak, M. S. *Befriending bumble bees: a practical guide to raising local bumble bees*. (University of Minnesota, 2007).
3. Strange, J. P. Nest initiation in three North American bumble bees (Bombus): Gyne number and presence of honey bee workers influence establishment success and colony size. *J. Insect Sci.* **10**, (2010).
4. R Core team. R Core Team. *R: A Language and Environment for Statistical Computing. R Foundation for Statistical Computing , Vienna, Austria. ISBN 3-900051-07-0, URL http://www.R-project.org/.* **55**, 275–286 (2015).
5. Bates, D., Mächler, M., Bolker, B. & Walker, S. Fitting linear mixed-effects models using lme4. *J. Stat. Softw.* **67**, 1–48 (2015).
6. Kuznetsova, A., Brockhoff, P. B. & Christensen, R. H. B. lmerTest Package: tests in linear mixed effects models. *J. Stat. Softw.* **82**, (2017).
7. Bartoń, K. *MuMIn*: *Multi-model inference*. v.1.43.15 (R package, 2019).
8. Hijmans, R. J., Cameron, S. E., Parra, J. L., Jones, P. G. & Jarvis, A. Very high resolution interpolated climate surfaces for global land areas. *Int. J. Climatol.* **25**, 1965–1978 (2005).
9. Langfelder, P. & Horvath, S. WGCNA: an R package for weighted correlation network analysis. *BMC Bioinformatics* **9**, 559 (2008).
10. Campbell-Staton, S. C., Bare, A., Losos, J. B., Edwards, S. V. & Cheviron, Z. A. Physiological and regulatory underpinnings of geographic variation in reptilian cold tolerance across a latitudinal cline. *Mol. Ecol.* **27**, 2243–2255 (2018).
11. Cheviron, Z. A., Connaty, A. D., Mcclelland, G. B. & Storz, J. F. Functional genomics of adaptation to hypoxic cold-stress in high-altitude deer mice: Transcriptomic plasticity and thermogenic performance. *Evolution* **68**, 48–62 (2014).
12. Robinson, M. D., McCarthy, D. J. & Smyth, G. K. edgeR: a Bioconductor package for differential expression analysis of digital gene expression data. *Bioinformatics* **26**, 139–140 (2010).
13. Ritchie, M. E. *et al.* limma powers differential expression analyses for RNA-sequencing and microarray studies. *Nucleic Acids Res.* **43**, e47–e47 (2015).
14. Vuong, Q. H. Likelihood ratio tests for model selection and non-nested hypotheses. *Econometrica* **57**, 307 (1989).
15. Kenward, M. G. & Roger, J. H. An improved approximation to the precision of fixed effects from restricted maximum likelihood. *Comput. Stat. Data Anal.* **53**, 2583–2595 (2009).
